# PeptideBERT: A Language Model Based on Transformers for Peptide Property Prediction

**DOI:** 10.1021/acs.jpclett.3c02398

**Published:** 2023-11-13

**Authors:** Chakradhar Guntuboina, Adrita Das, Parisa Mollaei, Seongwon Kim, Amir Barati Farimani

**Affiliations:** † Department of Electrical and Computer Engineering, Carnegie Mellon University, Pittsburgh, Pennsylvania 15213, United States; ¶ Department of Biomedical Engineering, Carnegie Mellon University, Pittsburgh, Pennsylvania 15213, United States; ∥ Department of Mechanical Engineering, Carnegie Mellon University, Pittsburgh, Pennsylvania 15213, United States; § Department of Chemical Engineering, Carnegie Mellon University, Pittsburgh, Pennsylvania 15213, United States; ⊥ Machine Learning Department, Carnegie Mellon University, Pittsburgh, Pennsylvania 15213, United States

## Abstract

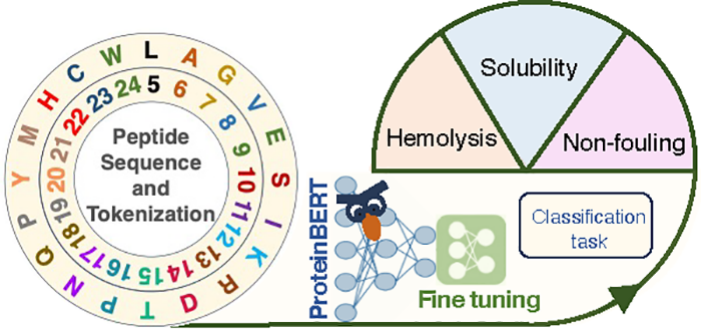

Recent advances in language models have enabled the protein modeling community with a powerful tool that uses transformers to represent protein sequences as text. This breakthrough enables a sequence-to-property prediction for peptides without relying on explicit structural data. Inspired by the recent progress in the field of large language models, we present PeptideBERT, a protein language model specifically tailored for predicting essential peptide properties such as hemolysis, solubility, and nonfouling. The PeptideBERT utilizes the ProtBERT pretrained transformer model with 12 attention heads and 12 hidden layers. Through fine-tuning the pretrained model for the three downstream tasks, our model is state of the art (SOTA) in predicting hemolysis, which is crucial for determining a peptide’s potential to induce red blood cells as well as nonfouling properties. Leveraging primarily shorter sequences and a data set with negative samples predominantly associated with insoluble peptides, our model showcases remarkable performance.

Peptides are organic molecules containing amino acids, ranging from only a few amino acids to numerous units that are joined together in ordered sequences.^1−6^ The length and arrangement of amino acids in a sequence govern a protein’s structural and biological properties.^7−10^ Consequently, the peptide sequence determines how the peptide engages with its environment and various molecules. For example, the peptides’ therapeutic properties such as hemolysis, fouling characteristics, and solubility^11−13^ are defined by sequences of amino acids. Hemolysis refers to the disintegration of red blood cells,^14^ and understanding its connection to the peptide’s amino acid sequence is vital to formulating safe and efficacious peptide-based treatments. Peptides that are fouling are less likely to adhere to or interact with molecules present in their environment.^15,16^ By exploring the influence of the peptide sequence on nonfouling properties, one can engineer the biocompatibility, durability, and overall effectiveness of designed biomaterials, medical devices, and drug delivery systems. Peptides’ solubility, which refers to the ability of a peptide to dissolve in a solvent, significantly affects their delivery and efficacy.^17^ Understanding and manipulating this sequence–structure–function relationship is crucial for peptide design in drug development and biomolecular engineering.^18^ Given the significance of mapping the sequence of the peptide to its properties, there have been many modeling attempts to perform this task. The quantitative structure–activity relationship (QSAR) models were previously used to build the relationship between the sequence and structural properties of chemical compounds.^19^ QSAR was used to predict the properties of several classes of peptides to sequences including inhibitory peptides,^20−22^ antimicrobial peptides,^23−25^ and antioxidant peptides.^26−28^ For solubility predictions, DSResol^29^ outperformed models such as DeepSol,^30^ SoluProt,^31^ and Protein-Sol^32^ with an accuracy of 75.1%. However, most of these models require the structure of the peptide, which makes it difficult to have access to a large variety of peptides. DSResol discerns extensive-range interaction information among amino acid k-mers utilizing dilated convolutional neural networks. MahLooL^33^ has comparable performance with respect to DSResol. MahLooL outperforms DSResol only for peptides of very short length (18–50), with an accuracy of 91.3%. MahLooL employs bidirectional long short-term memory (LSTM) networks to capture extensive sequence correlations. HAPPENN^34^ stands as a state-of-the-art (SOTA) model for predicting hemolytic activity, achieving an accuracy of 85.7%. HAPPENN employs normal features selected through support vector machines (SVMs) and an ensemble of random forests.

With the rise of transformers and large language models (LLMs),^35−37^ new deep learning architectures have emerged for modeling protein sequences since amino acid sequences can be considered as words and sentences similar to the language. Specifically, the attention mechanism of LLMs allows them to capture both immediate and intricate connections between elements of various types of textual data. As a result, it has initiated a revitalization in the field of bioinformatics since protein sequences, similar to languages, exhibit complex interactions among amino acids. Using LLM and transformers, we are now able to leverage advanced language modeling techniques to investigate the contributions of amino acids in the protein’s features.^38^ In this study and by taking advantage of transformers and pretraining, we developed PeptideBERT, a language model that predicts the peptide properties using only amino acid sequences as the input. By taking advantage of pretrained models such as ProtBert, we fine-tuned PeptideBERT to be able to predict the peptide’s properties (Figure 1). Pretrained models such as ProtBERT^39^ learned the protein sequence representation by being trained on massive protein sequences. We demonstrated that PeptideBERT can predict the hemolysis, nonfouling characteristics, and solubility of a given peptide using language models.

**Figure 1:**
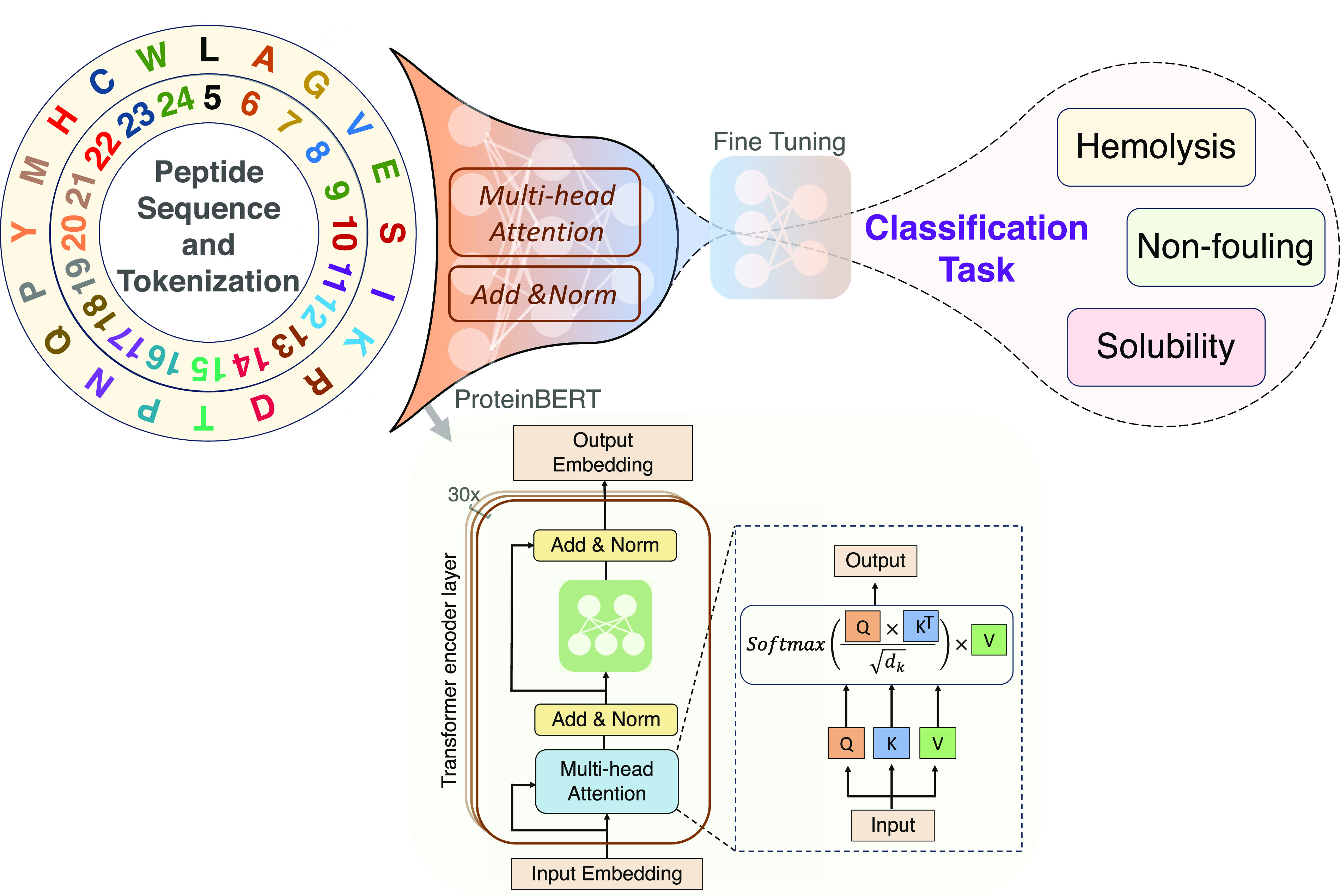
Model architecture of PeptideBERT. Peptide sequences are tokenized and subsequently processed through ProtBERT. Subsequently, a classification head of multilayer perceptrons (MLPs) is added for the fine-tuning process. The model is individually trained on three different classification downstream tasks: hemolysis, nonfouling, and solubility.

## Methods

**Figure 2:**
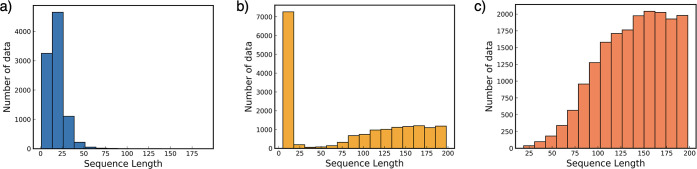
Sequence length of each peptide property data set: (a) hemolysis, (b) nonfouling, and (c) solubility.

The data sets employed for each specific task and their corresponding sequence length distributions are visually depicted in Figure 2. For the nonfouling data set, the length of sequences falls within the range of 2 to 20 residues. This particular data set focuses on comparatively shorter sequences, which are likely to capture specific characteristics relevant to the nonfouling property. In contrast, the data set utilized for the solubility task encompasses a broader spectrum of sequence lengths, spanning from 18 to 198 residues. This wide-ranging sequence length distribution is indicative of the diverse nature of sequences included in this data set, potentially accommodating a variety of structural and functional attributes. The use of data sets with distinct sequence length profiles highlights the tailored approach taken to address the unique requirements of each predictive task, further enhancing the model’s ability to capture and interpret the relevant information accurately.

The term hemolysis relates to the disruption of the membranes of red blood cells, which leads to a decrease in the lifespan of cells. It is essential to identify antimicrobial agents or peptides that do not cause hemolysis, as this ensures their safe and nontoxic use against bacterial infections. Antimicrobial peptides (AMPs) represent a collection of small peptides, recognized for their efficacy against bacteria, viruses, and fungi. Although these peptides exhibit limited bioavailability and short lifetimes, they possess distinct advantages over other categories of drugs or peptides including their notable specificity, selectivity, and minimal toxicity.^40,41^ However, distinguishing between peptides that cause hemolysis and those that do not is challenging because their main effects occur on the charged surface of bacterial cell membranes. Primarily, these peptides function as agents that modulate the immune response, induce apoptosis, and hinder cell proliferation. In recent times, there have been various endeavors to compile databases containing AMPs and to use computational techniques to categorize their hemolytic properties. In this study, the Database of Antimicrobial Activity and Structure of Peptides (DBAASPv3)^42^ was utilized for the hemolytic activity prediction model. The extent of activity is assessed by extrapolating measurements from dose–response curves to the point where 50% of red blood cells (RBCs) are lysed. Peptides with activity below 100 μg/mL are categorized as hemolytic.^33^ Each measurement is treated as an independent case, meaning that sequences can appear multiple times in the data set. The training data set comprises 9316 sequences, with 19.6% being positive (hemolytic) and 80.4% being negative (nonhemolytic). The sequences consist of only l- and canonical amino acids. It is worth noting that due to the inherent variability in experimental data, around 40% of observations contain identical sequences that are labeled as both negative and positive. For instance, a sequence such as “RVKRVWPLVIRTVIAGYNLYRAIKKK” has been found to exhibit both hemolytic and nonhemolytic behavior in two different laboratory experiments, resulting in two distinct training examples.^33^

The solubility data set consists of 18,453 sequences, with 47.6% being labeled as positives and 52.4% being labeled as negatives. These labels are based on information sourced from PROSO II.^43^ The solubility of the sequences was determined through a retrospective evaluation of electronic laboratory notebooks, which were part of a larger initiative known as the Protein Structure Initiative. The analysis involves tracking the sequences through various stages (such as selected, expressed, cloned, soluble, purified, crystallized), HSQC (heteronuclear single quantum coherence), structure determination, and submission to the Protein Data Bank (PDB).^44^ The categorization of peptides as soluble or insoluble is explained in PROSO II,^43^ achieved by contrasting their experimental status at two specific time points, September 2009 and May 2010. Specifically, those proteins that were initially insoluble in September 2009 and remained in the same insoluble state 8 months later were classified as insoluble.

The information employed to forecast resistance against nonspecific interactions (nonfouling) is gathered from this^24^ work. The positive data set comprises 3,600 sequences, while the negative examples are drawn from 13,585 sequences, yielding a distribution of 20.9% positives and 79.1% negatives. The negative data are drawn from insoluble and hemolytic peptides along with scrambled positives. To generate the scrambled negatives, sequences are chosen with lengths drawn from a range identical to that of their corresponding positive set. The residues for these sequences are chosen based on the frequency distribution observed in the solubility data set. The data set was compiled following the approach outlined in this^45^ work. A nonfouling peptide (considered to be a positive example) is defined following the methodology introduced by White et al.^45^ White et al. demonstrated that the amino acid frequencies on the exterior surfaces of proteins differ significantly, with this discrepancy becoming more pronounced in environments prone to protein aggregation such as the cytoplasm. They established that synthesizing self-assembling peptides adhering to this amino acid distribution and applying these peptides to surfaces yields nonfouling surfaces. This pattern was also observed within chaperone proteins, an area where mitigating nonspecific interactions is crucial.^46^

The provided data sets^33^ have been preprocessed by applying a custom encoding method. In this encoding, the integer representation of each of the 20 amino acids is given by its index in the following array (indexing starts from 1): [A, R, N, D, C, Q, E, G, H, I, L, K, M, F, P, S, T, W, Y, V]. For example, the sequence “A M N D V” is converted to 1 13 3 4 20.

Since we are using ProtBERT and since its tokenizer uses a different encoding process, to ensure compatibility, we first converted all of the data sets from integers to characters using reverse mapping and then converted them back to integers using ProtBERT’s encoding. Following the encoding procedure, we split each data set into three nonoverlapping subsets: a training set (to train the model) consisting of 81% of the data set, a validation set (for hyperparameter tuning) consisting of 9% of the data set, and a test set (to benchmark the model’s performance on unseen data) consisting of 10% of the data set. This specific train–validation–test split of 81%–9%–10% has been selected to ensure a proper comparison between our approach and the previous methodologies.^33^ Data augmentations, if any (such as in the solubility task), are then applied to the training set, while the validation and test sets remain unchanged.

Data augmentation is a technique commonly employed in machine learning, especially in scenarios where there is limited data or overfitting is a concern. Its primary purpose is to artificially increase the size and diversity of the training data set by introducing minor modifications to the original data. This not only helps in providing more training examples but also enhances the model’s ability to generalize, thus potentially improving its performance on unseen data.

For the solubility task, the necessity for data augmentation arose from the fact that our initial model’s performance was found to be suboptimal compared to the benchmarks set by earlier research in the field. As such, we experimented with the following data augmentation techniques on the solubility data set in order to improve the model’s classification accuracy for the task:

- random_replace: Randomly replace a given fraction of the unpadded protein sequence with random amino acids. For example, if the fraction is 0.1 and the protein sequence is “A M N D V E T R L H”, then the output will be something like “A M N D V E **M** R L H”.
- random_delete: Randomly delete a given fraction of the unpadded protein sequence. For example, if the fraction is 0.1 and the protein sequence is “A M N D V E T R L H”, then the output will be something like “A N D V E T R L H”.
- random_replace_with_A: Randomly replace a given fraction of the unpadded protein sequence with the amino acid “A”. For example, if the fraction is 0.1 and the protein sequence is “A M N D V E T R L H”, then the output will be something like “A M N **A** V E T R L H”.
- random_swap: Randomly swap a given fraction of adjacent pairs of amino acids in the unpadded protein sequence. For example, if the fraction is 0.1 and the protein sequence is “A M N D V E T R L H”, then the output will be something like “A M N D V E T **L R** H”.
- random_insertion_with_A: Randomly insert amino acid “A” into the unpadded protein sequence and subsequently increase its length by a given fraction. For example, if the fraction is 0.1 and the protein sequence is “A M N D V E T R L H”, then the output will be something like “A M N D V E T R L **A** H”.
- random_mask: Randomly mask or replace certain elements in the sequence with a [MASK] token. The mask token is typically chosen to represent missing or irrelevant information and is often assigned a specific integer value. For example, if the masking probability is 0.2, then about 20% of the elements in the sequence will be selected for masking. So, for a given protein sequence “A M N D V E T R L H”, after masking the selected elements with [MASK] token, the sequence becomes “A M **[MASK]** D **[MASK]** E **[MASK]** R L H”.

Regarding our choice of fraction for data augmentation, we initially started with a fraction of 0.2, hypothesizing that anything more substantial might introduce excessive alterations and noise into the data set. However, to strike a balance between data diversity and overaugmentation, we performed systematic experiments by progressively decreasing this fraction. After iterative testing, we settled on a fraction of 0.02 (for most of the techniques except for the random masking technique), which yielded the most significant improvement in model performance without introducing noticeable noise or adverse effects.

Applying these augmentations resulted in varying degrees of improvement in the model’s classification accuracy. The results are shown in Table 1. The best-performing augmentation was random_swap with a 0.843% increment in accuracy.

**Table 1:** Ablation Results for Different Augmentation Techniques for the Solubility Prediction^a^

Augmentations applied	Train set size	Accuracy(%)
random_replace(2%)	29892	68.694
random_delete(2%)	29892	68.814
random_replace_with_A(2%)	29892	68.573
random_swap(2%)	29892	70.018
random_insertion_with_A(2%)	29892	69.597
random_swap(2%), random_insertion_with_A(2%)	44838	68.453
random_swap(2%), random_insertion_with_A(1%)	44838	68.814
random_swap(3%)	29892	68.814
random_replace_with_A(2%), random_insertion_with_A(2%)	44838	69.054

a Baseline accuracy (without any augmentation) is 69.175%.

The architectural blueprint of PeptideBERT is shown in Figure 1. At its core, PeptideBERT uses the pretrained ProtBERT,^39^ a transformer model that consists of 12 attention heads and 12 hidden layers. Its design is influenced by the original BERT^36^ model. ProtBERT is pretrained on a massive corpus of protein sequences (UniRef100^47^) containing over 217 million unique protein sequences. During its pretraining phase, a masked language modeling (MLM) objective was employed. Here, 15% of the amino acids in sequences were masked, challenging the model to predict these hidden segments based on the surrounding context. Additionally, this pretraining was performed in a self-supervised manner using only raw protein sequences without any human-generated labels. The attention mechanism is a pivotal component of transformer architectures, designed to model dependencies in sequences irrespective of the distance between elements. At its core, the attention mechanism computes a weighted sum of input values (often termed “values” or V), where each weight indicates the relevance or “attention” a specific input should receive given a query. The weights are determined by calculating the dot product between the query (Q) and associated keys (K), followed by a softmax operation to ensure that the weights are normalized and that they sum to 1. This allows the transformer to focus more on certain parts of the input while attending less to others. In the context of natural language processing, for instance, this can mean focusing on specific words in a sentence that are more pertinent to understanding the context or meaning of another word. The multihead attention architecture further enhances this by enabling the model to attend to multiple parts of the input simultaneously, capturing diverse relationships in the data. By doing so, transformers can learn intricate patterns and long-range dependencies,^48^ making them particularly effective for a plethora of sequence-based tasks. Such a transformative encoder structure in ProtBERT allows the model to glean context-sensitive representations of amino acids, treating each protein sequence akin to a “document”. ProtBERT is followed by a regression head, which is a fully connected neural network that takes the output of ProtBERT and maps it to a continuous value. The regression head is a single fully connected layer with 480 nodes. The output of the regression head is passed through a sigmoid function to ensure that the output is between 0 and 1. The output of the sigmoid function is then thresholded to 0.5 to obtain the final binary prediction. The optimal architecture for the regression head was determined by performing a series of experiments, the results of which are discussed in the results section.

For each task, a separate model was fine-tuned on the corresponding data set. The model was trained using the AdamW optimizer of the binary cross-entropy loss function with an initial learning rate of 0.00001 and a batch size of 32. The model was trained for 30 epochs. The ReduceLROnPlateau scheduler was employed to reduce the learning rate by a factor of 0.1 if the validation accuracy did not improve for four epochs. The model was trained on a single NVIDIA GeForce GTX 1080Ti GPU with 16GB of memory. The training time and optimal hyperparameters for each task are outlined briefly in the Supporting Information.

As seen in the bottom of Figure 1, the tokenized residues enter the embedding matrix, and each becomes a vector representation (or embedding) subsequently entering the attention matrix. However, before entering the embedding matrix, the classification token ([CLS] token) is typically added at the beginning of the sequence in the tokenization process. During training, the model learns to use the [CLS] token as a representation that encapsulates the holistic information for the entire input sequence. Since [CLS] token is always placed at a consistent position at the beginning of the sequence, the model associates this specific token (and its position) with the task of aggregating information from the entire sequence. Thus, [CLS] token is often used as a representation of the entire input sequence. To effectively visualize PeptideBERT’s understanding of various peptide sequences and its classification competence, we extracted the [CLS] token embeddings of each peptide sequence from the final hidden state and visualized it with t-distributed stochastic neighbor embedding (t-SNE).^49^ The t-SNE algorithm performs dimensionality reduction by evaluating similarities between pairs of data points in a high-dimensional space and creating a Gaussian joint probability distribution. In parallel, a comparable probability distribution is produced using the Student’s t-distribution. The method then aims to reduce the Kullback–Leibler divergence between these two distributions. This reduction process naturally pulls similar data points closer and pushes dissimilar ones apart, thus enabling the grouping of similar data points.

The performance and efficiency of our proposed model, PeptideBERT, are shown through a comprehensive analysis of its achieved outcomes. The solubility prediction task presented a significant challenge due to the presence diverse range of lengths of sequences within the data set. Given the complexity and variability of peptide sequences, this particular prediction task demanded a tailored approach to enhance the model’s performance. To address this challenge and improve the model’s ability to generalize across a wide spectrum of sequences, we employed an augmentation strategy. Table 1 outlines the various augmentation techniques we applied and their impact on the solubility prediction accuracy. This approach aimed to expose the model to a more comprehensive array of sequence variations, effectively expanding its learning capacity. By performing augmentation on the data set, we were able to introduce an increased diversity of sequence patterns and characteristics, enabling the model to better capture the underlying features that influence solubility prediction. Random replace at a rate of 2% led to an accuracy of 68.694%, while random delete, also at 2%, yielded an accuracy of 68.814%. The introduction of random replace with A at 2% demonstrated an accuracy of 68.573%. Notably, the random swap augmentation at 2% showcased an improved accuracy of 70.018%.

Similarly, random insertion with A at 2% exhibited an accuracy of 69.597%. A combination of random swap and random insertion with A, both at 2%, achieved an accuracy of 68.453% on a larger training set of 44,838 samples. It is interesting that employing a lower rate (1%) of random insertion with A in conjunction with random swap maintained an accuracy of 68.814%. The application of random swap at 3% resulted in an accuracy of 68.814%, akin to the accuracy produced by replacing with A and inserting with A, both at 2%. Table 2 provides a comprehensive comparison of classification accuracies across various models, including our novel ProtBERT-based model across three distinct prediction tasks. For the nonfouling prediction task, our PeptideBERT model demonstrated exceptional performance, achieving an accuracy of 88.365%, significantly surpassing the accuracy of 82.0% attained by the embedding + LSTM approach.

**Table 2:** Classification Accuracy Comparison of Previous Methods and our ProtBERT-Based Approach on Each of the Three Prediction Tasks

Approach	Task	Accuracy(%)
PeptideBERT (ours)	nonfouling	**88.365**
Embedding + LSTM	nonfouling	82.0
PeptideBERT (ours)	hemolysis	**86.051**
embedding + Bi-LSTM	hemolysis	84.0
UniRep + logistic regression	hemolysis	82.0
UniRep + random forests	hemolysis	84.0
HAPPENN^34^	hemolysis	85.7
HLPpred-Fuse^50^	hemolysis	-
one-hots + RNN^51^	hemolysis	76.0
PeptideBERT (ours) (with augmentation)	solubility	70.018
PeptideBERT (ours) (without augmentation)	solubility	69.175
embedding + Bi-LSTM	solubility	70.0
PROSO II^43^	solubility	71.0
DSResSol (1)^29^	solubility	**75.1**

Moreover, the PeptideBERT model outperformed the other models in the hemolysis task, achieving an accuracy of 86.051%, while the embedding + Bi-LSTM and UniRep + logistic regression approaches achieved 84.0% and 82.0% accuracies, respectively. This showcases the robustness of our model in predicting hemolytic properties. In the solubility prediction task, our PeptideBERT model demonstrated competitive results. With data augmentation, it achieved a predictive accuracy of 70.018%, while without augmentation it attained an accuracy of 69.17%. Comparatively, the Embedding + Bi-LSTM and PROSO II methods achieved 70.0% and 71.0% accuracies, respectively. These findings highlight the effectiveness of our PeptideBERT-based approach, which consistently achieved higher accuracies across all three prediction tasks, both with and without data augmentation, showcasing its potential to enhance predictive capabilities in diverse bioinformatics applications.

**Figure 3:**
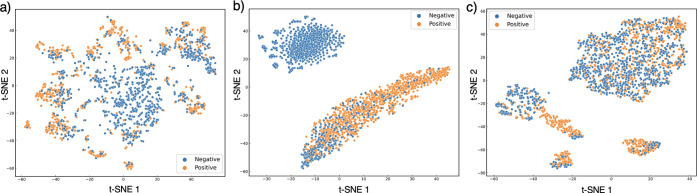
t-SNE visualization of peptide properties: (a) hemolysis, (b) nonfouling, and (c) solubility. The [CLS] token embedded from the last hidden state of PeptideBERT is visualized after dimensionality reduction.

The visualization results of [CLS] token embeddings, which have a size of 480, are shown in Figure 3. The t-SNE visualization clearly illustrates that peptides with similar properties (represented by identical color markers) are clustered together. Furthermore, the result indicates the model’s capability of classifying peptides solely based on their sequence information and that the [CLS] token within the embeddings has effectively captured the distinguishing features of individual peptides. From the observed patterns, the model appears to segregate peptides into two distinct groups (outer and inner for the hemolysis data set), meaning that the binary classification downstream tasks were valid for fine-tuning the PeptideBERT. The model’s errors are also noticeable; for example, the blue dots positioned in the bottom right of the nonfouling t-SNE (Figure 3(b)) mean that the misclassified peptides are negative nonfouling. Comparing all three plots, the hemolysis (Figure 3(a)) and nonfouling (Figure 3(b)) t-SNE show clear classification while the solubility t-SNE has a relatively large number of errors aligning with the accuracy results from the fine-tuning procedure (Table 2).

In this paper, we introduced three sequence-based classifiers aimed at predicting peptide hemolysis, solubility, and resistance to nonspecific interactions (nonfouling). These classifiers demonstrate competitive performance in comparison with the latest state-of-the-art models. The PeptideBERT model for the hemolysis prediction task is designed to predict a peptide’s capacity to cause red blood cell lysis. It is tailored for peptides spanning 1 to 190 residues and involves l- and canonical amino acids. PeptideBERT provides state-of-the-art sequence-based hemolysis predictions with an accuracy of 86.051%. This accuracy suggests that the model can reliably identify peptides with hemolytic potential, contributing to better decision making in peptide design and application. It is important to note that the training data set (refer to the Data sets section for a brief outline) for hemolysis prediction comprises peptide sequences that possess antimicrobial or clinical significance. While this targeted approach certainly boosts the model’s performance within these particular domains, prudent consideration is needed when extending its predictions to a wider array of peptides. The fact that our hemolytic model is specifically designed for peptides with lengths ranging from 1 to 190 residues reflects an important consideration that peptide length can significantly influence their behavior, including interactions with cells or molecules. By tailoring the model to this specific length range, we take into account the structural variations that can arise in different peptide lengths. Our nonfouling model also provides state-of-the-art predictions with an accuracy of 88.365% for the nonfouling task, which is designed to predict the ability of a peptide to resist nonspecific interactions. It is also noteworthy that the model yielded nearly comparable performance (85.99%), after removing the overlapping sequences labeled as both positive and negative from the data set (refer to the Supporting Information section for a more detailed explanation). The training data for the nonfouling task primarily consist of shorter sequences in the range of 2–20 residues. The data set employed for this task consists of instances of negative examples that are predominantly associated with insoluble peptides, which could lead to an increase in accuracy if only soluble peptides are compared.^33^ Our predictive model achieves an accuracy of 70.018% with augmentations for the solubility task. This accuracy can be primarily attributed to the challenges involved in predicting solubility in cheminformatics.^33^

In this work, we developed a language model called PeptideBERT to predict various peptide properties including hemolysis, solubility, and nonfouling. Our model takes advantage of pretrained models that learned the representation of protein sequences. Using PeptideBert, we demonstrate a hemolysis predictor and a nonfouling predictor that outperforms existing state-of-the-art models. The performance of these classifiers demonstrates their potential utility in the field of peptide research and applications. Notably, the model for hemolysis prediction exhibits robust predictive capabilities, offering valuable insights into the potential of peptides to cause red blood cell lysis. However, it is important to acknowledge the focused nature of its training data set, which primarily encompasses sequences with antimicrobial or clinical relevance. As such, while these classifiers show promising results, a prudent approach involves considering the context and potential limitations when their predictions are applied to a broader range of peptides. The competitive results compared to state-of-the-art models underline the progress made in predictive peptide modeling using language models. It suggests that the newly introduced models are not just novel but also effective in capturing relevant features that contribute to peptide behavior. The predictive capabilities of these classifiers hold promise for diverse applications, ranging from drug design to bioengineering. Accurate predictions of properties such as hemolysis, solubility, and resistance to nonspecific interactions can aid in identifying peptides with desired characteristics for therapeutic or functional purposes.

## Data Availability

The necessary code (including scripts to download the data sets) used in this study can be accessed here: https://github.com/ChakradharG/PeptideBERT.

## Supporting Information Available

Optimal hyperparameters and training time, additional ablation studies for the solubility and hemolysis tasks, confusion matrix and Fisher’s exact test, and reproducibility (PDF)
