# Supplementary material for: PeptideBERT: A Language Model Based on Transformers for Peptide Property Prediction
Source: J Phys Chem Lett. 2023 Nov 13;14(46):10427–34. doi: 10.1021/acs.jpclett.3c02398 (PMC10683064; doi:10.1021/acs.jpclett.3c02398)
Supplement: Supplementary file 1 — jz3c02398_si_001.pdf [file jz3c02398_si_001.pdf]

PeptideBERT: A Language Model based on Transformers for Peptide Property Prediction Supporting Information

Chakradhar Guntuboina,[†,‡] Adrita Das,[¶,‡] Parisa Mollaei,[§] Seongwon Kim,[‖] and Amir Barati Farimani[*,§,¶,⊥]

†*Department of Electrical and Computer Engineering, Carnegie Mellon University, 15213, USA*

‡*Authors Contributed Equally*

¶*Department of Biomedical Engineering, Carnegie Mellon University, 15213, USA*

§*Department of Mechanical Engineering, Carnegie Mellon University, 15213, USA*

‖*Department of Chemical Engineering, Carnegie Mellon University, 15213, USA*

⊥*Machine Learning Department, Carnegie Mellon University, 15213, USA*

E-mail: barati@cmu.edu

Optimal Hyperparameters and Training Time

The effectiveness of our model is evident from its training time across various prediction tasks as highlighted in Table 1. For the*Nonfouling* task, the model required 58.28 minutes for training, while for*Hemolysis* prediction, the training time was slightly longer at 69.28 minutes. The *Solubility* prediction task demanded more extensive training, taking 116.42 minutes to converge. The hyperparameters that played a pivotal role in shaping our model's performance, are illustrated in Table 2. The optimal hyperparameter values were determined

Table 1: Time taken to train the model on each of the 3 prediction tasks

Task	Training time (minutes)
Nonfouling	58.28
Hemolysis	69.28
Solubility	116.42

Table 2: Hyperparameters along with their optimal values used to train PeptideBERT

Hyperparameter	Optimal value
Initial LR	$1.0 * 10^{-5}$
Batch Size	32
Number of Attention Heads	12
Number of Hidden Layers	12
Hidden Size	480
Hidden Layer Dropout	0.15
LR Scheduler (factor)	0.1
LR Scheduler (Patience)	4

after a careful fine-tuning process. An initial learning rate (Initial LR) of $1.0 * 10^{-5}$ was determined to be the optimal learning rate, managing a trade-off between rapid convergence and preventing overfitting. The model performed well with a batch size of 32, and the model configuration consisted of 12 attention heads and 12 hidden layers, each comprising 480 hidden units. To prevent overfitting, a dropout rate of 0.15 was employed between hidden layers. The learning rate scheduler, with a reduction factor of 0.1, along with the patience of 4, contributed to a more stable convergence process.

Additional ablation studies for the Solubility and Hemolysis tasks

In order to assess the effectiveness of different data augmentation techniques in improving the performance of our model for the Solubility task, we conducted some additional ablation studies as outlined in Table 3. The ablation study involved applying *Random Masking* to the training set, at different masking probabilities. The results revealed a pattern of diminishing accuracy as the augmentation level increased. Specifically, when applying a random masking probability of 0.15, our model achieved an accuracy of 68.784%. A slight decrease in accuracy was observed when masking probability of 0.20 was applied(67.863%). Further decrease in

Table 3: Ablation results of other augmentation techniques applied for the *Solubility* task

Augmentations applied	Train set size	Accuracy(%)
random_mask(15%)	29524	68.784
random_mask(20%)	29524	67.863
random_mask(30%)	29524	65.894

Table 4: Results of the ablation studies for the *Hemolysis* task

Hyperparameters	Value	Accuracy(%)
num_hidden_layers,hidden_dim,num_attention_heads	12,480,12	83.010
num_hidden_layers,hidden_dim,num_attention_heads	48,560,24	78.865

Table 5: Results of the ablation studies for the *Hemolysis* task (After removing the overlapping sequences in the hemolysis dataset)

Hyperparameters	Hidden layers	Value	Acc(%)
num_hidden_layers,hidden_dim,num_attention_heads	1 hidden layer	48,240,12	85.59
num_hidden_layers,hidden_dim,num_attention_heads	2 hidden layers	48,560,24	79.537

accuracy was observed when the masking probability was further increased to 0.30. These findings indicate the trade-off between data augmentation and model performance. Results of the ablation studies shown in Table 4 shed light on how varying different hyperparamters influence performance outcomes.

The configuration with 12 hidden layers, a hidden dimension of 480, and 12 attention heads demonstrated an accuracy of 83.010%. On the other hand, the second configuration, characterized by a more complex architecture with 48 hidden layers, a larger hidden dimension of 560, and 24 attention heads, achieves a still commendable accuracy of 78.865%.

This indicates that while increased model depth and attention head count can potentially introduce more intricate representations in the model architecture, there exists a threshold beyond which the advantages of having more complex representations might plateau or even diminish.

In Table 55, we present the results of ablation studies conducted for the Hemolysis task after preprocessing the hemolysis dataset to remove overlapping sequences (sequences labeled both as positive and negative).

The ablation studies focused on investigating the impact of varying hyperparameters and model architecture on the task's accuracy. Two key architectures were explored specifically focused on the structure of the hidden layers in the model: (1) a single additional hidden layer was introduced on top of the pre-trained BERT-based layers, achieving an accuracy of 85.599% ; (2) two consecutive hidden layers were added, yielding an accuracy of 79.537%.

Confusion Matrix and Fisher's Exact Test

In order to analyze our model's performance at a greater depth, we computed the True Positive count (TP), False Positive count (FP), True Negative count (TN), and False Negative count (i.e., the confusion matrix) for each subset (train, validation, test) of each dataset (Non-Fouling, Hemolysis, Solubility). These are shown in Fig 1. Further, we used these counts (TP, FP, TN, FN) to perform Fisher's Exact test (2-sided) and compute the p-value for each of the 9 aforementioned subsets. The results for these are shown in Table 6.

Table 6: P-values from Fisher's Exact Test

Dataset	Train	Validation	Test
Hemolysis	0.0	$1.287480665169691 * 10^{-32}$	$3.3815652647983235 * 10^{-46}$
Non-Fouling	0.0	$1.9428605594595628 * 10^{-110}$	$5.754059799916193 * 10^{-129}$
Solubility	0.0	$1.262458469748561 * 10^{-41}$	$1.781274978743949 * 10^{-67}$

Reproducibility

To address concerns related to the reproducibility of our results, we conducted additional rounds of experiments and recorded their outcomes. We repeated the training procedure for all tasks three separate times, maintaining the same conditions and parameters across all iterations. Table7 showcases the outcomes of these three trials.

Upon analysis, it is evident that the results across the three repetitions demonstrate a consistent pattern, thereby reinforcing the reproducibility and robustness of our initial findings. Minor variations that may appear across iterations can be attributed to the inherent

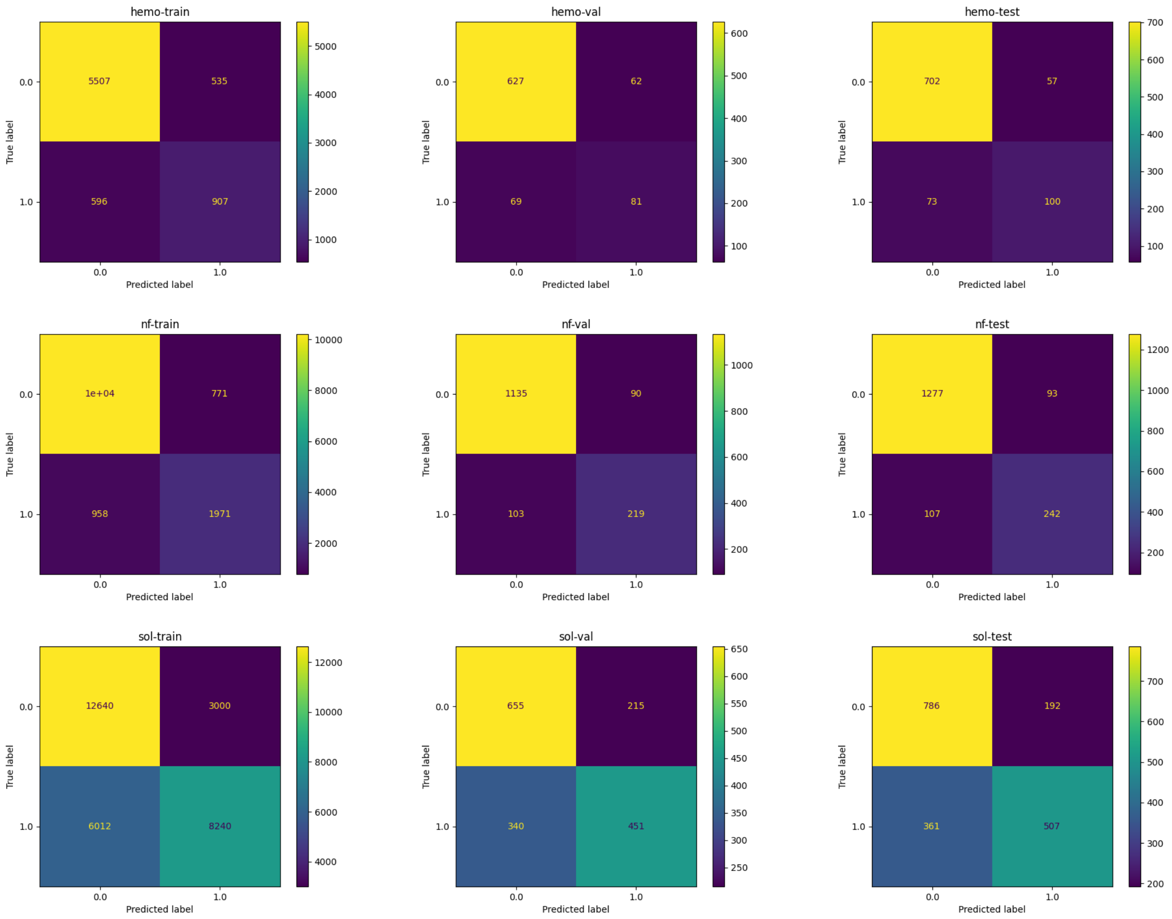

Figure 1: (From top left, going left-right, top-bottom) Confusion Matrix for Hemolysis-train, Hemolysis-validation, Hemolysis-test, Non-Fouling-train, Non-Fouling-validation, Non-Fouling-test, Solubility-train, Solubility-validation, Solubility-test datasets

Table 7: Reproducibility Assessment: Results of three independent runs of the training procedure for each dataset

Dataset	Run 1	Run 2	Run 3
Hemolysis	85.73%	84.335%	84.013%
Non-Fouling	87.842%	88.133%	87.9%
Solubility	68.147%	69.88%	68.96%

stochasticity of the training process, which is a common characteristic in deep learning models.

By providing these additional results, we hope to instill greater confidence in the reliability and validity of our presented findings.
